# Profile of soluble cell adhesion molecules as potential biomarkers in the cardiac stages of chronic Chagas disease

**DOI:** 10.3389/fimmu.2025.1541860

**Published:** 2025-03-07

**Authors:** Victor Vaitkevicius-Antão, Bruno Almeida Silva, Michelle da Silva Barros, Cíntia Nascimento da Costa-Oliveira, Byannca de Carvalho Torreão, Ana Carla da Silva, Silvia Marinho Martins, Cristina Carrazone, Wilson Oliveira, Carolina de Araújo Medeiros, Michelle Christiane da Silva Rabello, Virginia Maria Barros de Lorena

**Affiliations:** ^1^ Laboratory of Imunoparasitology, Aggeu Magalhães Institute, Oswaldo Cruz Foundation, Recife, Pernambuco, Brazil; ^2^ Departament of Medicina Tropical, Federal University of Pernambuco, Recife, Brazil; ^3^ Chagas Disease and Heart Failure Outpatient Clinic, Pronto Socorro Cardiológico Universitário de Pernambuco (PROCAPE), University of Pernambuco, Recife, Brazil; ^4^ Nursing School Nossa Senhora das Graças (FENSG), University of Pernambuco, Recife, Brazil

**Keywords:** cardiomyopathy, prognostic, selectins, *Trypanosoma cruzi*, VCAM-1

## Abstract

Chagas cardiomyopathy is the most severe chronic manifestation and leading cause of mortality in the disease. Chronic inflammation, resulting from persistent infection by *T. cruzi*, leads to continuous immune system activation in patients with Chagas disease. The knowledge of immunological aspects can be important for the identification of biomarkers capable of indicating parasitological cure or clinical progression provides for physician’s valuable tools for improved clinical evaluation. Soluble cell adhesion molecules (sCAMs) have been applied in other disease like potential biomarkers. Thus, the aim of this study was to evaluate the levels of soluble cell adhesion molecules in chronic patients with different stages of Chagas heart disease progression. Sera from 303 individuals, classified according to cardiac involvement and left ventricular dysfunction, were used for cell adhesion molecules quantification (sVCAM-1, sP-selectin, sE-selectin and sL-selectin) and it was measured using the Cytometric Bead Array. We found that sCAMs demonstrated good performance in differentiating patients in the indeterminate phase from negative individuals or those in the mild cardiac phase, as well as patients with mild from those with severe cardiomyopathy, and cardiac patients non-infected *versus* infected (severe cardiomyopathy). Therefore, sCAMs may serve as potential biomarkers for the diagnosis and prognosis of chronic cardiac patients with Chagas disease.

## Introduction

Chagas disease, also known as American trypanosomiasis, is a protozoan infection endemic to Latin America, with high morbidity, and is caused by the hemoflagellate protozoan *Trypanosoma cruzi* (1). The disease is characterized by two phases: acute and chronic. The acute phase, which can last up to four months after infection, is characterized by intense parasitaemia and strong inflammatory response. The chronic phase follows the acute phase, can be asymptomatic or symptomatic, and the clinical progression of individuals is unpredictable (2, 3).

Most chronic patients (~70%) remain asymptomatic for decades and are considered to have the indeterminate form of the disease, as they do not show signs of cardiac or digestive progression (4). Approximately 30% to 45% of chronic patients may develop the cardiac form, characterized by Chagas cardiomyopathy, the most severe chronic manifestation and the leading cause of mortality in the disease (5).

Chronic Chagas cardiomyopathy is defined as a dilated cardiomyopathy associated with extensive fibrosis, leading to the progressive loss of contractile function of the organ, which may culminate in heart failure (6, 7). This damage to the myocardium and intramural nervous system occurs due to the presence of *T. cruzi* and inflammatory response, leading to necrosis and fibrosis, resulting in hemodynamic and cardiovascular repercussions (8).

In addition, several factors such strain variability, cellular tropism, the host’s immune response, and genetic background, directly contribute to cardiac pathogenesis in Chagas disease (9, 10). The immunological aspects that help explain why patients evolve into different clinical forms of the disease are addressed by several theories, which, while conflicting, may also be interconnected, providing insight into the immunopathogenesis of the disease’s clinical manifestations.

The persistence of *T. cruzi* in the host results in chronic inflammation, leading to continued immune system activation in patients with Chagas disease. This persistence of the parasite in the chronic phase triggers a cellular immune response that causes extensive tissue damage without repair (11). High levels of Th1-profile cytokines (IFN-γ and TNF) are involved in the progression of the cardiac form, while IL-10 is associated with the indeterminate form, protecting against the development of symptomatic chronic forms (12, 13). However, factors leading asymptomatic chagasic patients to develop cardiomyopathy are still unknown (14).

Chemical mediators of the inflammatory process stimulate the expression of cell adhesion molecules (CAMs), which participate in the recruitment of leukocytes to the infection site. Selectins are transmembrane molecules involved in the rolling and margination of leukocytes. They are expressed on the cell surface and divided into three types. E-selectin is present in endothelial cells and is expressed after stimulation by inflammatory cytokines (15. P-selectin is found in platelets as well as on endothelial cells and is expressed after stimulation by thrombin and histamine (16). L-selectin is expressed in most circulating leukocytes (17). In turn, VCAM-1 (vascular cell adhesion molecule-1) is expressed in endothelial cells in response to inflammatory interleukins and TNF, interacting with integrins (molecules present on the surfaces of leukocytes). VCAM-1 plays a role in the later stages of rolling by to facilitate cell adhesion to the endothelium, enabling subsequent transmigration (18).

These CAMs are expressed on the cell surface and are subsequently cleaved by metalloproteases, releasing their soluble form into the bloodstream, making their quantification possible (19). Some studies have been investigating the importance of CAMs in heart diseases (20, 21), as well as their role in diseases (22), such as *T. cruzi* infection (23, 24).

Among individuals infected with the parasite, fewer than 10% are diagnosed, and this diagnosis usually occurs in the chronic phase (25). Due to the lack of prognostic markers, treatment should be administered to all potentially affected patients (those in the undetermined stages or with initial symptoms) (26), with approximately 1% of those infected receiving treatment (25). Treatment when administrated in the chronic phase have the effectiveness reduced, however even when treated in the acute phase, there are reports of the development of chronic heart disease (27).

Thus, the identification of biomarkers capable of indicating parasitological cure or clinical progression provides for physician’s valuable tools for improved clinical evaluation and decision-making (28). Moreover, these new biomarkers could also be instrumental in the search for safer and more effective therapeutic options, as well as enabling closer and more accurate monitoring of treated patients (26). Given the above, it is believed that CAMs could be used as prognostic biomarkers in the chronic form of Chagas disease. Therefore, this study aimed to evaluate the levels of soluble cell adhesion molecules in chronic patients with different stages of Chagas heart disease progression.

## Material and methods

### Study population

Sera from 303 individuals from the Chagas Disease and Heart Failure Outpatient Clinic - PROCAPE - Universidade de Pernambuco were analysed. These individuals were classified into different groups according to the results of diagnostic tests (conventional and recombinant ELISA, IgG anti- *T. cruzi*) based to the II Brazilian Consensus on Chagas Disease (4). Also, clinical exams (echocardiogram, electrocardiogram, X-ray) were performed. Individuals were excluded if they presented: esophageal or colon dilation, digestive complaints (such as dysphagia or constipation), leukocyte alterations, pacemaker implantation, hypo – or hyperthyroidism, autoimmune diseases, cancer or any other systemic diseases. Patients who had received blood transfusions or organ transplants were also excluded.

Thus, patients who tested positive in the serological test were classified into the following groups: asymptomatic infected individuals (I-A, n = 102), individuals with mild cardiac involvement (I-MC, n = 70), and individuals with severe cardiac involvement (I-SC, n = 87). In addition, non-reactive individuals in the serological test were also analyzed for comparison and classified based on the presence (non-infected cardiac individuals, NI-C, n = 22) or absence (asymptomatic non-infected individuals, NI-A, n = 22) of any cardiac problem.

The cardiopathy of the infected was classified based on left ventricular dysfunction and manifestations of heart failure, according to the I Latin American Guideline for the Diagnosis and Treatment of Chagas Cardiomyopathy (8). Group I-A patients were classified as stage A (asymptomatic), group I-MC patients as stage B1 (mild electrocardiographic and echocardiographic alterations with preserved ventricular function), and group I-SC patients as stage C (ventricular dysfunction). From the percentage of left ventricular ejection fraction (LVEF), it was possible to characterize cardiac function (**Table 1**).

**Table 1:** Characterization of chagasic and non-chagasic individuals.

	NI-A	NI-C	I-A	I-MC	I-SC	TOTAL
Number of individuals	22	22	102	70	87	303
Female	17	7	59	48	47	178
Male	5	15	43	22	40	125
Age*	50,8	59,5	50,8	58,9	58,5	55,7
LVEF%*	–	48.1^c,d,e^	66.3^b,e^	63.7^b,e^	38.7^b,c,d^	–

NI-A (non-infected asymptomatic); NI-C (non-infected cardiac); I-A (infected asymptomatic); I-MC (infected mild cardiac); I-SC (infected severe cardiac). Statistical significance at p < 0.05 are represented by superscript letters ‘b’, ‘c’, ‘d’ and ‘e’ for comparation with NI-C, I-A, I-MC and I-SC respectively. * (Mean values). Kruskal-Wallis test and Dunn’s multiple comparation test.

### Molecules quantification

The levels of soluble cell adhesion molecules (sVCAM-1/CD106, sP-selectin/CD62P, sE-selectin/CD62E, and sL-selectin/CD62L) were measured using the Cytometric Bead Array (CBA) Flex system (Becton Dickinson, BD CBA Human, San Jose, CA, USA), according to the manufacturer’s instructions, with adaptations as required. The beads were acquired within 24 hours using the BD FACSCalibur flow cytometer. The analyses were performed using BD FCAP Array Software version 3.0.1.

### Data analysis

The data derived from FCAP Array analyses were processed using GraphPad Prism 8 (GraphPad Software Inc., 2018, San Diego, CA, USA). To evaluate the normality of the data, the Shapiro-Wilk test was applied. Based on the distribution characteristics of the samples, variance analyses were performed using the Kruskal-Wallis test for non-parametric data or One-Way ANOVA for parametric data, followed by *post-hoc* multiple comparison tests (Dunn’s for non-parametric and Tukey’s for parametric analyses). When comparing two groups, parametric data were analyzed using the T-test, while non-parametric data were evaluated with the Mann-Whitney test (unpaired) or Wilcoxon test (paired). Statistical significance was determined at a threshold of p < 0.05.

Correlations between the molecules were examined using the Spearman test for non-parametric data and the Pearson test for parametric data. The strength of correlations was represented by the correlation coefficient (r) and classified as follows: 0 - 0.3 (negligible), 0.3 - 0.5 (weak), 0.5 - 0.7 (moderate), 0.7 - 0.9 (strong), and 0.9 - 1.0 (very strong) (29).

To assess the capacity of sCAMs to discriminate among the defined groups, Receiver Operating Characteristic (ROC) curve analysis was performed. The evaluation included the determination of optimal cut-off points and the calculation of the area under the curve (AUC), which served as a measure of test performance. AUC values were interpreted as follows: 0.5 - 0.6 (very poor), 0.6 - 0.7 (poor), 0.7 - 0.8 (fair), 0.8 - 0.9 (good), and above 0.9 (excellent) (30). Also, a cut-off was established through of the means concentrations of the groups analyzed, to distinguish individuals as “high” and “low” secretors of sCAMs (31). Fisher’s exact test was used to compare the frequencies of high sCAMs secretors among the different groups, with significance considered at p < 0.05.

### Ethical considerations

Individuals included in the study were invited to participate and sign an Informed Consent Form, authorizing the use of the collected material for scientific purposes. This study is part of the project “Prospecting for Biomarkers of Clinical Evolution and Etiological Treatment in Chagas Disease,” which has been approved by the Research Ethics Committee of the Aggeu Magalhães Institute CAAE: 20049919.5.0000.51.90.

## Results

**Figure 1:**
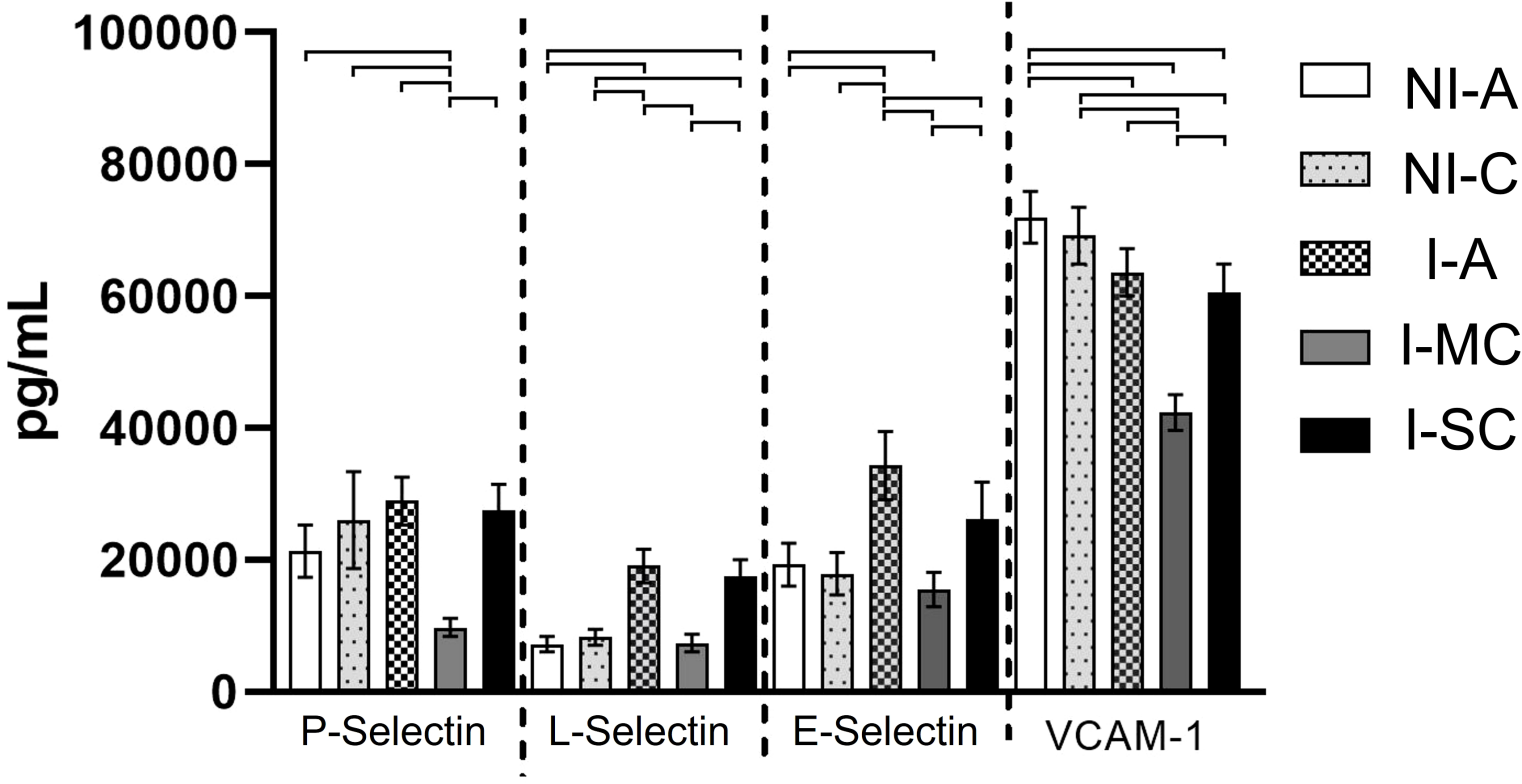
Average concentrations of soluble cell adhesion molecules in the different study groups. NI-A (non-infected asymptomatic); NI-C (non-infected cardiac); I-A (infected asymptomatic); I-MC (infected mild cardiac); I-SC (infected severe cardiac). ┌┐Statistically significant (p < 0.05). Error bars (Confidence Interval of 95%). Analysis of Variance: Kruskal-Wallis test and Dunn’s multiple comparation test.

Our data shows variations in levels of sCAMs in the different groups analysed. We found that patients in group I-MC demonstrated lower mean levels compared to other chronic patients. Selectins showed higher mean levels in group I-A compared to non-infected individuals (NI-A and NI-C). In contrast, sVCAM-1 levels were lower in the chronic patient groups than in asymptomatic non-infected individuals, while patients with cardiac infections (I-MC and I-SC) had lower levels than non-infected cardiac patients (NI-C) (**Figure 1**).

A significant moderate positive correlation was observed between LVEF and sE-selectin (r = 0.513; p < 0.001) in the I-SC group. These results indicate that patients with lower LVEF tend to have lower sE-selectin levels in the most severe form of chronic chagasic cardiopathy.

**Figure 2:**
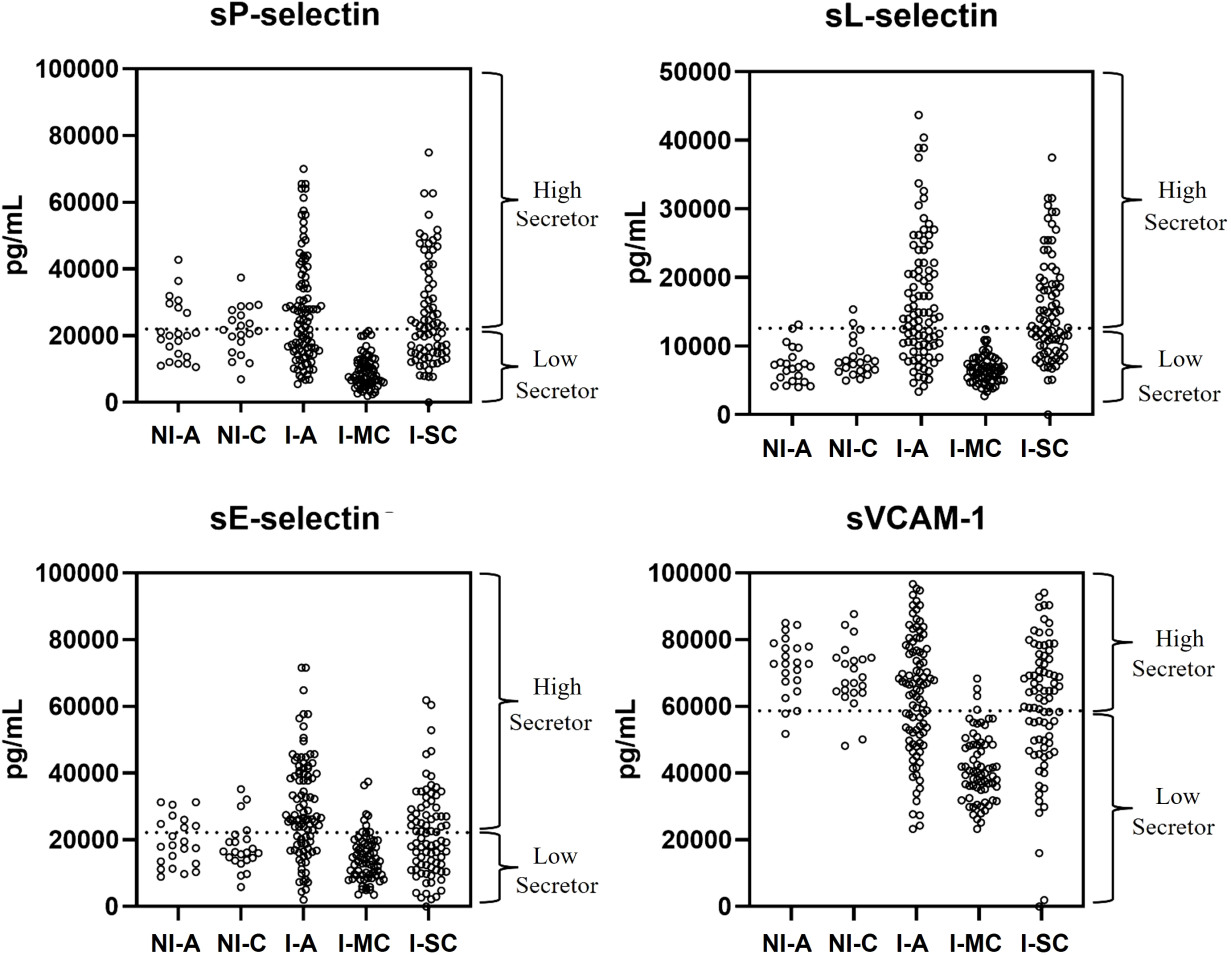
Representative scatter of individual molecule concentrations by group, differentiating high and low producers based on the adopted cut-off (mean concentration of all individuals per molecule). NI-A (non-infected asymptomatic); NI-C (non-infected cardiac); I-A (infected asymptomatic); I-MC (infected mild cardiac); I-SC (infected severe cardiac).

By adopting a concept of “high” and “low” sCAM secretors, and establishing a cut-off based on the average concentrations of all individuals, we investigated the frequency of secretion of these molecules between the groups (**Figure 2**). All individuals in group I-MC were classified as low secretors of sP-selectin and sL-selectin, showing a statistically significant difference compared to all other groups (p < 0.0001). It was also observed that while most non-infected individuals were classified as high secretors of sVCAM-1, most of these individuals were classified as low secretors of sL-selectin. The percentage frequencies of high secretors for all groups are summarized in **Table 2**.

**Table 2:** Relative frequency of individuals in each group, classified as high secretors of soluble cell adhesion molecules.

	Cut-off (pg/ml)	NI-A	NI-C	I-A	I-MC	I-SC
sP-selectin	22.044,29	31,82%	45,00%	53,68%^d^	0,00% ^a,b,c,e^	53,25%^d^
sL-selectin	12.602,62	4,55%	9,09%	59,78% ^a,b,d^	0,00% ^c,e^	54,32% ^a,b,d^
sE-selectin	22.202,22	36,36%	18,18%	68,75% ^a,b,d,e^	11,59% ^a,c,e^	46,34% ^a,b,c,d^
sVCAM-1	58.681,18	86,36%	90,91%	61,46% ^a,b^	5,80% ^a,b,c,e^	58,75% ^a,b,d^

NI-A (non-infected asymptomatic); NI-C (non-infected cardiac); I-A (infected asymptomatic); I-MC (infected mild cardiac); I-SC (infected severe cardiac). Statistical significance at p < 0.05 are represented by superscript letters ‘a’, ‘b’, ‘c’, ‘d’ and ‘e’ for comparation with NI-A, NI-C, I-A, I-MC and I-SC respectively. Fisher’s exact test.

**Figure 3:**
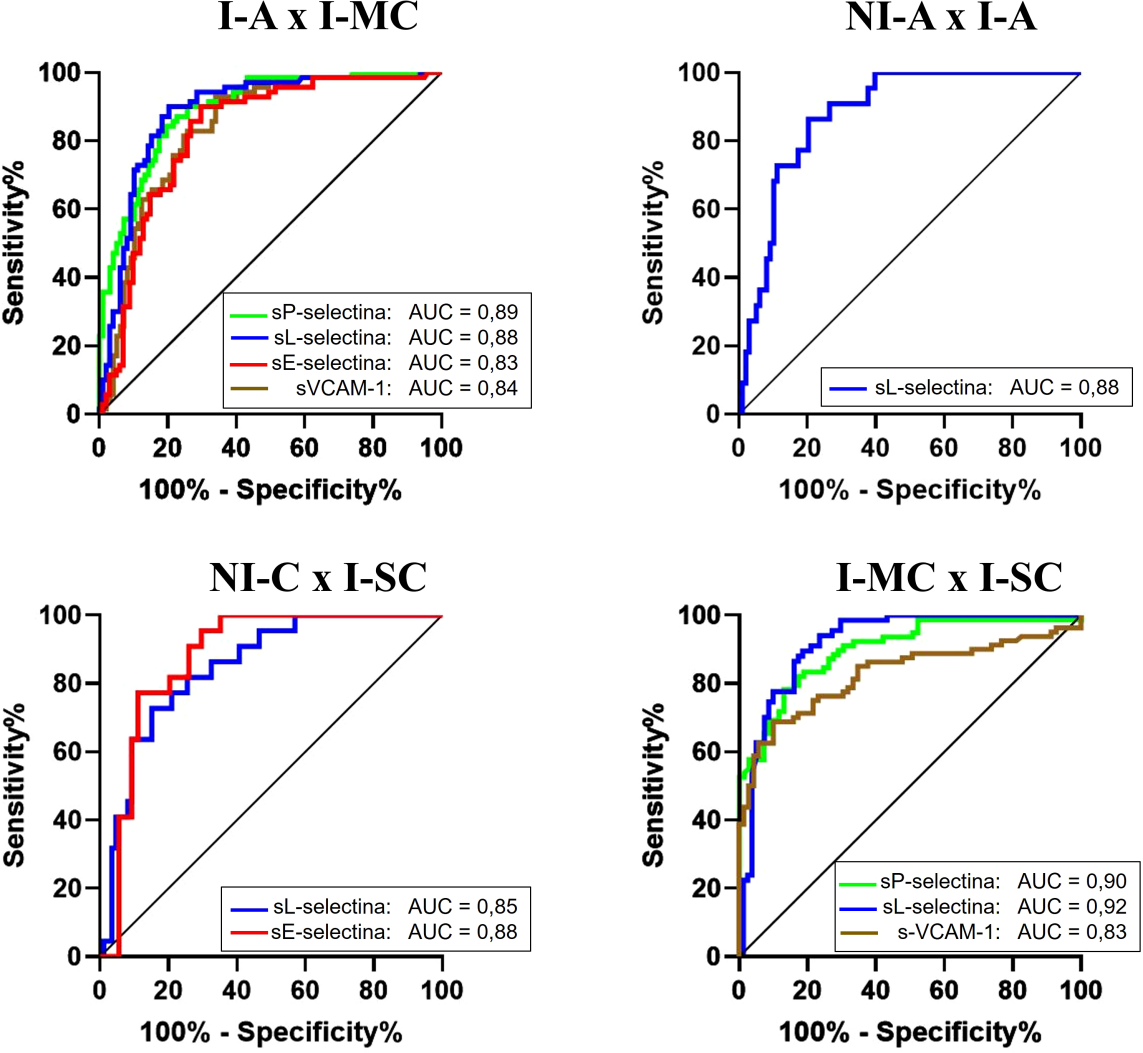
Comparison of ROC curves of molecules between different groups. NI-A (non-infected asymptomatic); NI-C (non-infected cardiac); I-A (infected asymptomatic); I-MC (infected mild cardiac); I-SC (infected severe cardiac). AUC (area under the curve).

Moreover, by plotting the ROC curve for the sCAMs and analyzing the AUC, we can highlight the ability of the sCAMs to discriminate between distinct groups based on the cut-off points adopted for each molecule. **Figure 3** illustrates the sCAMs with an AUC above 0.8.

The analysis of I-A and I-MC patient groups, the sCAMs exhibited good AUC values (sP-selectin = 0.89; sL-selectin = 0.88; sE-selectin = 0.83; sVCAM-1 = 0.84), demonstrating that these molecules can differentiate between chronic asymptomatic (I-A) and the initial cardiac phase forms (I-MC). When comparing NI-A and I-A, sL-selectin stands out with an AUC of 0.88, indicating its ability to differentiate between infected and non-infected individuals, despite both groups lacking clinical signs. In the comparison between the NI-C and I-SC groups, significance was observed with sL-selectin and sE-selectin (AUC = 0.85 and 0.88, respectively), highlighting the ability of these two molecules to differentiate between infected and non-infected individuals, even though both have heart disease. To differentiate between the I-MC and I-SC groups, sP-selectin and sVCAM-1 presented AUCs of 0.90 and 0.83, respectively. The molecule that stood out the most was sL-selectin, which demonstrated excellent accuracy (AUC = 0.92).

## Discussion

Cardiac fibrosis is a hallmark of Chagas cardiomyopathy, and its progression over time is associated with decreased left ventricular systolic function (32). The characterization of heart disease based on the I Latin American Guideline for the Diagnosis and Treatment of Chagas Cardiomyopathy facilitates the study of cardiac forms with and without ventricular dysfunction (8). The investigation of various molecules as potential biomarkers for Chagas disease has already been conducted (33), however, it does not address the different stages of Chagas heart disease. Thus, our study focuses on analyzing the immune response through soluble adhesion molecules in chronic patients, with differentiation based on the cardiac stage.

In our study, we observed a distinct variation in the levels of sCAMs across the different stages of Chagas disease. In chronic patients transitioning from the indeterminate form (I-A group) to mild chronic Chagas cardiomyopathy (I-MC group), sCAM levels exhibited a significant decrease. However, as the disease progressed to more severe stages of cardiomyopathy (I-SC group), these levels increased again, approaching concentrations observed in the indeterminate group. A similar profile has been reported for chemokines such as CCL5 and CXCL10 in patients with indeterminate and chronic Chagas heart disease (34).

The initial reduction in sCAM levels observed between the indeterminate and mild cardiac groups may be linked to the inflammatory response associated with the Th1 profile. This response, while critical for controlling *Trypanosoma cruzi* infection, can lead to excessive production of nitric oxide and reactive oxygen species. These reactive molecules are known to suppress the expression of adhesion molecules involved in cellular migration and tissue infiltration (35). To control the inflammation, anti-inflammatory cytokines (such as IL-10 and TGF-β) are expressed to regulate this heightened inflammatory response. Vasconcelos et al. (36) observed that chagasic patients with mild cardiomyopathy exhibit a stronger anti-inflammatory response (IL-10) compared to indeterminate form and severe cardiomyopathy patients. Similarly, Melo et al. (37) reported a high frequency of IL-10 genes in mild cardiomyopathy patients. This scenario of suppression may explain the decline in sCAMs levels during the early stages of cardiac involvement.

As the disease progresses to severe cardiomyopathy, the inflammatory process becomes increasingly intense. Teixeira et al. (38) provide additional insights into the immunopathological mechanisms underlying these observations. They describe how the Th1-dominated response in Chagas disease promotes the release of inflammatory cytokines and chemokines. These mediators drive the recruitment of immune cells to the myocardium, consequently increasing sCAMs levels and leading to necrosis and apoptosis of cardiomyocytes. This tissue damage perpetuates the inflammatory cycle and exacerbates disease progression, facilitating the transition from I-MC to I-SC patients.

This progression results in elevated levels of sCAMs, like demonstrated in our data, which show an elevation in sCAMs levels in severe cardiac patients (I-SC group) compared to those with mild cardiac involvement (I-MC group). This trend aligns with findings by Yin et al. (39), who observed that levels of sP-selectin, sVCAM-1, and sICAM-1 increased with the severity of cardiac heart failure in patients with various etiologies unrelated to Chagas disease. Although the study does not focus specifically on Chagas cardiomyopathy, it highlights how worsening cardiac dysfunction correlates with elevated levels of sCAMs. Therefore, the results presented (sCAMs levels) along with other studies on oxidative stress and inflammatory mediators, offer a comprehensive explanation for the variations in sCAM levels across the stages of Chagas disease.

We also observed that infection by *T. cruzi* (in indeterminate chronic patients) favors the modulation of the studied molecules when compared to uninfected individuals, with higher concentrations of selectins and lower concentrations of sVCAM-1. Our findings corroborate those of Panagiotidou et al. (40), who demonstrated the ability of the parasite to induce the production of selectins, using them as mediators for transendothelial transmigration and allowing the parasite to target specific tissues.

By adopting the concept of “high producers” and “low producers” (41), we were able to establish a relationship between the studied molecules and the patient’s clinical status, combined with accuracy analysis (ROC curve). We observed the good ability of sCAMs to differentiate between the indeterminate and mild cardiac stages (I-A vs. I-MC), suggesting their potential use as clinical biomarker for predicting early cardiac changes. In addition to sL-selectin demonstrating good accuracy in distinguishing infected from uninfected asymptomatic individuals (I-A vs. NI-A), being considered a complementary toll for diagnosis of infection, however further studies are carried out.

Cardiac markers, such as Troponin and CKMB, which increase with the severity of cardiac damage, are already in use. However, they are insufficient to distinguish Chagas heart disease from other cardiac conditions (42). Therefore, our study stands out by identifying molecules (sL-selectin and sE-selectin) that show good accuracy in differentiating non-infected heart disease patients from those with severe chronic Chagas heart disease (NI-C vs. I-SC). Additionally, LVEF% demonstrated a positive correlation with sE-selectin, supporting the potential of this molecule as a biomarker for differentiating Chagasic from non-Chagasic heart disease.

Furthermore, studies that perform a stratification cardiac severity are scarce, making it difficult to effectively assess the progression of cardiac involvement in the disease. By addressing this gap in the literature, our findings reveal that sVCAM-1, sP-selectin, and sL-selectin demonstrate strong potential in differentiating mild and severe heart disease (I-MC vs I-SC).

This study provides scientific evidence on the profile of cell adhesion molecules across different stages of cardiac progression in the chronic phase of Chagas disease. However, further research is needed to understand the mechanisms of sCAMs, particularly through future longitudinal studies, which may help predict the clinical progression of the disease. Therefore, continued investigation of sCAMs in the context of Chagas disease will offer valuable insights for the scientific community.

## Conclusion

CAMs demonstrated good performed well in differentiating patients in the indeterminate phase from negative individuals, as well as from those in the mild cardiac phase. Left ventricular ejection fraction combined with sE-selectin proved to be a good indicator for distinguishing between uninfected and infected cardiac individuals for improved prediction. Finally, sL-selectin demonstrated excellent accuracy in differentiating infected patients with mild from those with severe cardiomyopathy. Therefore, we suggest that sCAMs may serve as potential biomarkers for the diagnosis and prognosis of chronic cardiac patients with Chagas disease.

## Data Availability

The original contributions presented in the study are included in the article/Supplementary Material. Further inquiries can be directed to the corresponding author.

## References

[1] Schmunis GA Yadon ZE. Chagas disease: A Latin American health problem becoming a world health problem. Acta Trop. (2010) 115:14–21. doi: 10.1016/j.actatropica.2009.11.003 19932071

[2] Lidani KCF Andrade FA Bavia L Damasceno FS Beltrame MH Messias-Reason IJ. Chagas disease: from discovery to a worldwide health problem. Front Public Health. (2019) 7:166. doi: 10.3389/fpubh.2019.00166 31312626 PMC6614205

[3] Rassi A Rassi A Marin-Neto JA. Chagas disease. Lancet. (2010) 375:1388–402. doi: 10.1016/S0140-6736(10)60061-X 20399979

[4] Dias JCP Ramos AN Jr Gontijo ED Luquetti A Shikanai-Yasuda MA Coura JR. II Consenso Brasileiro em Doença de Chagas, 2015: Aspectos gerais da epidemiologia da Doença de Chagas, com especial atenção ao Brasil. Epidemiol Serv Saúde. (2016) 25:7–86. doi: 10.5123/S1679-49742016000500002 27869914

[5] Pérez-Molina JA Molina I. Chagas disease: the Latin American perspective of the disease. Lancet. (2018) 391:82–94. doi: 10.1016/S0140-6736(17)31612-4 28673423

[6] Echavarría NG Echeverría LE Stewart M Gallego C Saldarriaga C. Chagas disease: chronic Chagas cardiomyopathy. Curr Probl Cardiol. (2020) 45:100507. doi: 10.1016/j.cpcardiol.2019.100507 31983471

[7] Marin-Neto JA Cunha-Neto E Maciel BC Simoes MV. Pathogenesis of chronic Chagas heart disease. Circulation. (2007) 115:1109–23. doi: 10.1161/106.624296 17339569

[8] Andrade JP Marin-Neto JA Paola AA Vilas-Boas F Oliveira GM Bacal F. I Diretriz Latino-Americana para o Diagnóstico e Tratamento da Cardiopatia Chagásica. Arq Bras Cardiol. (2011) 97:1–48. doi: 10.1590/S0066-782X2011000600002 21952638

[9] Alvarado-Arnez LE Batista AM Alves SM Melo G Lorena VMB Cardoso CC. Single nucleotide polymorphisms of cytokine-related genes and association with clinical outcome in a Chagas disease case-control study from Brazil. Mem Inst Oswaldo Cruz. (2018) 113:e180164. doi: 10.1590/0074-02760180164 PMC5961924 29768622

[10] Dutra WO Gollob KJ. Current concepts in immunoregulation and pathology of human Chagas disease. Curr Opin Infect Dis. (2008) 21:287–92. doi: 10.1097/QCO.0b013e3282fc9d50 PMC3322114 18448974

[11] Gutierrez FRS Guedes PMM Gazzinelli RT Silva JS. The role of parasite persistence in pathogenesis of Chagas heart disease. Parasite Immunol. (2009) 31:673–85. doi: 10.1111/j.1365-3024.2009.01157.x 19825107

[12] Dutra WO Menezes CA Villani FN Reis D Gollob KJ. Cellular and genetic mechanisms involved in generation of protective and pathogenic immune responses in human Chagas disease. Mem Inst Oswaldo Cruz. (2009) 104:208–18. doi: 10.1590/S0074-02762009000900027 PMC3285444 19753476

[13] Gomes JAS Bahia-Oliveira LM Rocha MO Martins-Filho OA Gazzinelli G Correa-Oliveira R. Evidence that development of severe cardiomyopathy in human Chagas disease is due to a Th1-specific immune response. Infect Immun. (2003) 71:1185–93. doi: 10.1128/IAI.71.3.1185-1193.2003 PMC148818 12595431

[14] Cutshaw MK Sciaudone M Bowman NM. Risk factors for progression to chronic Chagas cardiomyopathy: a systematic review and meta-analysis. Am J Trop Med Hyg. (2023) 108:791–800. doi: 10.4269/ajtmh.22-0630 36848894 PMC10076993

[15] Souza Jd Sousa J Hirai KE Silva LM Fuzii HT Dias LB. E-selectin and P-selectin expression in endothelium of leprosy skin lesions. Acta Trop. (2015) 149:227–31. doi: 10.1016/j.actatropica.2015.06.002 26051909

[16] Khew-Goodall Y Butcher CM Litwin MS Newlands S Korpelainen Ei Noack LM. Chronic expression of P-selectin on endothelial cells stimulated by the T-cell cytokine, interleukin-3. Blood. (1996) 87:1432–8. doi: 10.1182/blood.v87.4.1432.bloodjournal8741432 8608233

[17] Ivetic A Hoskins Green HL Hart SJ. L-selectin: A major regulator of leukocyte adhesion, migration and signaling. Front Immunol. (2019) 10:1–22. doi: 10.3389/fimmu.2019.01068 31139190 PMC6527602

[18] Foster CA. VCAM-1/α4-integrin adhesion pathway: therapeutic target for allergic inflammatory disorders. J Allergy Clin Immunol. (1996) 98:270–7. doi: 10.1016/s0091-6749(96)70075-1 8977536

[19] Santos JC Cruz MS Bortolin RH Oliveira KM Araujo JNG Duarte VHR. Relationship between circulating VCAM-1, ICAM-1, E-selectin and MMP9 and the extent of coronary lesions. Clinics. (2018) 73:e203. doi: 10.6061/clinics/2018/e203 29846413 PMC5960074

[20] Demerath E Towne B Blangero J Siervogel RM. The relationship of soluble ICAM-1, VCAM-1, P-selectin and E-selectin to cardiovascular disease risk factors in healthy men and women. Ann Hum Biol. (2001) 28:664–78. doi: 10.1080/03014460110048530 11726042

[21] Troncoso MF Ortiz-Quintero J Garrido-Moreno V Sanhueza-Olivares F Guerrero-Moncayo A Chiong M. VCAM-1 as a predictor biomarker in cardiovascular disease. Biochim Biophys Acta Mol Basis Dis. (2021) 1867:166170. doi: 10.1016/j.bbadis.2021.166170 34000374

[22] Mousa SA. Cell adhesion molecules: potential therapeutic & diagnostic implications. Mol Biotechnol. (2008) 38:33–40. doi: 10.1007/s12033-007-0072-7 18095189

[23] Laucella SA Segura EL Riarte E Sosa E. Soluble platelet selectin (sP-selectin) and soluble vascular cell adhesion molecule-1 (sVCAM-1) decrease during therapy with benznidazole in children with indeterminate form of Chagas’ disease. Clin Exp Immunol. (1999) 118:423–7. doi: 10.1046/j.1365-2249.1999.01070.x PMC1905450 10594562

[24] Michailowsky V Silva NM Rocha CD Vieira LQ Lannes-Vieira J Gazzinelli RT. Intercellular adhesion molecule 1 deficiency leads to impaired recruitment of T lymphocytes and enhanced host susceptibility to infection with *trypanosoma cruzi* . J Immunol. (2004) 173:463–70. doi: 10.4049/jimmunol.173.1.463 15210806

[25] Chaves GC Abi-Saab-Arrieche M Rode J Mechali D Reis PO Alves RV. Estimación de la demanda de medicamentos antichagásicos: una contribución para el acceso en América Latina. Rev Panam Salud Publica. (2017) 41:e45. doi: 10.26633/RPSP.2017.45 28614468 PMC6612731

[26] Cortes-Serra N Losada-Galvan I Pinazo MJ Fernandez-Becerra C Gascon J Alonso-Padilla J. State-of-the-art in host-derived biomarkers of Chagas disease prognosis and early evaluation of anti-Trypanosoma cruzi treatment response. Biochim Biophys Acta Mol Basis Dis. (2020) 1866:165758. doi: 10.1016/j.bbadis.2020.165758 32169507

[27] Antunes AF Maduro SG Pereira BVM Barbosa MG Guerra JA Ferreira JM. Chronic heart disease after treatment of oral acute chagas disease. Arq Bras Cardiol. (2016) 107:184–6. doi: 10.5935/abc.20160108 PMC5074072 27627643

[28] Pinazo MJ Thomas MC Bua J Perrone A Schijman AG Viotti RJ. Biological markers for evaluating therapeutic efficacy in Chagas disease, a systematic review. Expert Rev Anti Infect Ther. (2014) 12:479–96. doi: 10.1586/14787210.2014.899150 24621252

[29] Mukaka MM. Statistics corner: a guide to appropriate use of correlation coefficient in medical research. Malawi Med J. (2012) 24:69–71. 23638278 PMC3576830

[30] Polo TCF Miot HA. Aplicações da curva ROC em estudos clínicos e experimentais. J Vasc Bras. (2020) 19:e20200186. doi: 10.1590/1677-5449.200186 34211533 PMC8218006

[31] Lorena VM Lorena IM Braz SC Melo AS Melo MF Melo MG. Cytokine levels in serious cardiopathy of Chagas disease after *in vitro* stimulation with recombinant antigens from *Trypanosoma cruzi* . Scand J Immunol. (2010) 72:529–39. doi: 10.1111/j.1365-3083.2010.02462.x 21044127

[32] Santos JB Gottlieb I Tassi EM sCAMargo GC Atie J Xavier SS. Fibrose Cardíaca e Mudanças Evolutivas na Função Ventricular Esquerda em Pacientes com Cardiopatia Chagásica Crônica. Arq Bras Cardiol. (2021) 117:1081–90. doi: 10.36660/abc.20200268 PMC8757166 34644785

[33] Keating SM Deng X Fernandes F Cunha-Neto E Ribeiro AL Adesina B. Inflammatory and cardiac biomarkers are differentially expressed in clinical stages of Chagas disease. Int J Cardiol. (2015) 199:451–9. doi: 10.1016/j.ijcard.2015.07.040 PMC4868386 26277551

[34] Souza-Silva TG Neves EGA Koh C Teixeira-Carvalho A Araújo SS Nunes MDCP. Correlation of blood-based immune molecules with cardiac gene expression profiles reveals insights into Chagas cardiomyopathy pathogenesis. Front Immunol. (2024) 15:1338582. doi: 10.3389/fimmu.2024.1338582 38390336 PMC10882095

[35] Basso B. Modulation of immune response in experimental chagas disease. World J Exp Med. (2013) 3:1. doi: 10.5493/wjem.v3.i1.1 24520540 PMC3905588

[36] Vasconcelos RHT Azevedo EAN Diniz GTN Cavalcanti MGAM Oliveira W Morais CNL. Interleukin-10 and tumour necrosis factor-alpha serum levels in chronic Chagas disease patients. Parasite Immunol. (2015) 37:376–9. doi: 10.1111/pim.2015.37.issue-7 25728555

[37] Melo AS Lorena VMB Braz SCM Docena C Gomes YM. IL-10 and IFN-γ gene expression in chronic Chagas disease patients after *in vitro* stimulation with recombinant antigens of Trypanosoma cruzi. Cytokine. (2012) 58:207–12. doi: 10.1016/j.cyto.2012.01.008 22325340

[38] Teixeira AR Hecht MM Guimaro MC Sousa AO Nitz N. Pathogenesis of Chagas’ disease: parasite persistence and autoimmunity. Clin Microbiol Rev. (2011) 24:592–630. doi: 10.1128/CMR.00063-10 21734249 PMC3131057

[39] Yin WH Chen JW Jen HL Chiang MC Huang WP Feng AN. The prognostic value of circulating soluble cell adhesion molecules in patients with chronic congestive heart failure. Eur J Heart Fail. (2003) 5:507–16. doi: 10.1016/s1388-9842(03)00009-6 12921812

[40] Panagiotidou S Anastasiou M Alcaide P Perrin MA. *Trypanosoma cruzi* exploits E- and P-Selectins to migrate across endothelial cells and extracellular matrix proteins. Infect Immun. (2021) 89:e0017821. doi: 10.1128/IAI.00178-21 34228487 PMC8445167

[41] Vitelli-Avelar DM Sathler-Avelar R Teixeira-Carvalho A Pinto Dias JC Gontijo ED Faria AM. Strategy to assess the overall cytokine profile of circulating leukocytes and its association with distinct clinical forms of human Chagas disease. Scand J Immunol. (2008) 68:516–25. doi: 10.1111/j.1365-3083.2008.02164.x 18803607

[42] Okamoto EE Sherbuk JE Clark EH Marks MA Gandarilla O Galdos-Cardenas G. Biomarkers in *Trypanosoma cruzi*-infected and uninfected individuals with varying severity of cardiomyopathy in Santa Cruz, Bolivia. PloS Negl Trop Dis. (2014) 8:e3227. doi: 10.1371/journal.pntd.0003 25275382 PMC4183477

